# A New Panel-Based Next-Generation Sequencing Method for ADME Genes Reveals Novel Associations of Common and Rare Variants With Expression in a Human Liver Cohort

**DOI:** 10.3389/fgene.2019.00007

**Published:** 2019-01-31

**Authors:** Kathrin Klein, Roman Tremmel, Stefan Winter, Sarah Fehr, Florian Battke, Tim Scheurenbrand, Elke Schaeffeler, Saskia Biskup, Matthias Schwab, Ulrich M. Zanger

**Affiliations:** ^1^Dr. Margarete Fischer-Bosch-Institute of Clinical Pharmacology, Stuttgart, Germany; ^2^Medical School, University of Tübingen, Tübingen, Germany; ^3^CeGaT GmbH, Tübingen, Germany; ^4^Praxis für Humangenetik Tübingen, Tübingen, Germany; ^5^Department of Clinical Pharmacology, University Hospital Tübingen, Tübingen, Germany; ^6^Department of Pharmacy and Biochemistry, University of Tübingen, Tübingen, Germany

**Keywords:** ADME, next generation sequencing, pharmacogenomics, eQTL analysis, rare variants

## Abstract

We developed a panel-based NGS pipeline for comprehensive analysis of 340 genes involved in absorption, distribution, metabolism and excretion (ADME) of drugs, other xenobiotics, and endogenous substances. The 340 genes comprised phase I and II enzymes, drug transporters and regulator/modifier genes within their entire coding regions, adjacent intron regions and 5′ and 3′UTR regions, resulting in a total panel size of 1,382 kbp. We applied the ADME NGS panel to sequence genomic DNA from 150 Caucasian liver donors with available comprehensive gene expression data. This revealed an average read-depth of 343 (range 27–811), while 99% of the 340 genes were covered on average at least 100-fold. Direct comparison of variant annotation with 363 available genotypes determined independently by other methods revealed an overall accuracy of >99%. Of 15,727 SNV and small INDEL variants, 12,022 had a minor allele frequency (MAF) below 2%, including 8,937 singletons. In total we found 7,273 novel variants. Functional predictions were computed for coding variants (*n* = 4,017) by three algorithms (Polyphen 2, Provean, and SIFT), resulting in 1,466 variants (36.5%) concordantly predicted to be damaging, while 1,019 variants (25.4%) were predicted to be tolerable. In agreement with other studies we found that less common variants were enriched for deleterious variants. *Cis*-eQTL analysis of variants with (MAF ≥ 2%) revealed significant associations for 90 variants in 31 genes after Bonferroni correction, most of which were located in non-coding regions. For less common variants (MAF < 2%), we applied the SKAT-O test and identified significant associations to gene expression for *ADH1C* and *GSTO1*. Moreover, our data allow comparison of functional predictions with additional phenotypic data to prioritize variants for further analysis.

## Introduction

Genetic variation in genes that function in the absorption, distribution, metabolism, and elimination (ADME) of drugs contributes significantly to the interindividual variability in efficacy and toxicity of numerous drugs from practically all therapeutic categories. In the past half century, pharmacogenetic research has unraveled many clinically meaningful associations between germline genetic variants and pharmacokinetic or drug response phenotypes (Meyer, 2004; Zanger and Schwab, 2013; Alfirevic and Pirmohamed, 2017). Clinical implementation of this knowledge is currently being pursued worldwide by several consortia (Caudle et al., 2013; Dunnenberger et al., 2015; Relling and Evans, 2015; Cecchin et al., 2017; Swen et al., 2018). For example, the Clinical Pharmacogenetics Implementation Consortium (CPIC) has so far issued 65 dosing guidelines for 38 drugs and 15 relevant genes (October 2018^1^). Until recently, pharmacogenetics has mainly focused on common genetic variants, which can be relatively easily assessed for association with pharmacokinetic or drug response phenotypes. However, a considerable proportion of genetic variability remains unexplained even for well-studied genes like CYP2D6, as recently shown by twin studies (Matthaei et al., 2015). Currently, it is widely assumed that rare deleterious variants fill this gap and contribute significantly to functional variability, which is further supported by the fact that rare variants are enriched for deleterious alleles due to purifying selection (1000 Genomes Project Consortium et al., 2012; Lek et al., 2016; Ingelman-Sundberg et al., 2018). Indeed, with the increasing availability of next-generation-sequencing (NGS) technology, several studies explored genetic variability of pharmacologically relevant “pharmacogenes” and revealed large numbers of rare variants, most of which were previously unknown (Tennessen et al., 2012; Fujikura et al., 2015; Han et al., 2016; Kozyra et al., 2016; Hovelson et al., 2017; Schärfe et al., 2017). For statistical reasons it is intrinsically more difficult to investigate the functional significance of rare variants as compared to common variants, especially regarding pharmacogenetic phenotypes, for which studies including relevant phenotypic data are essentially lacking. On the other hand, *in vitro* testing of thousands of variants is currently prohibitive for time and financial reasons. Current hopes to integrate rare variants into clinical pharmacogenomics therefore rely mainly on computational prediction tools, many of which are publically available (Ingelman-Sundberg et al., 2018; Zhou et al., 2018a). Computational predictions of “damaging” or “loss-of-function” (LOF) versus “tolerable” (TOL) functionality performed on ADME rare variants detected in genetic screens indicated that up to 30% of drug response variability could be due to rare variants and that likely every patient carries at least one “actionable” pharmacogenetic variant (Crosslin et al., 2015; Ji et al., 2016). However, data on the validity of functional prediction are scarce and their performance as well as the true contribution of rare variants to pharmacogenetics variability remains unclear, especially since current predictive algorithms rely largely on principles of evolutionary conservation, which may be more appropriate in the context of disease than for drug metabolism and response.

In this study we have developed a panel-based NGS pipeline for comprehensive sequence analysis of 340 ADME genes comprising all major genes known to be involved in phase 1 and phase 2 drug metabolism, drug transport and its regulation, as well as numerous additional genes of potential interest in this context. We applied our ADME NGS panel on genomic DNA from 150 human liver samples that we have previously genotyped by other methods and for which comprehensive mRNA expression data and some additional ADME phenotypes are available. This allowed us to directly compare genotype with expression for common and rare variants, unraveling numerous novel associations and potential candidates. In addition, we performed functional prediction for subsets of variants and exemplarily compared these with hepatic phenotype. This type of analysis, which has rarely been done, should be helpful to improve functional prediction and allow to prioritization of interesting rare variants for further analysis.

## Materials and Methods

### Patient DNA and Liver Samples

Liver tissues and corresponding blood samples were previously collected from patients of White European descent undergoing liver surgery at the Department of General, Visceral, and Transplantation Surgery (A. K. Nuessler, P. Neuhaus, Campus Virchow, University Medical Center Charité, Humboldt University Berlin, Germany) (Klein et al., 2012). The study protocol was approved by the ethics committees of the medical faculties of the Charité, Humboldt University, and the University of Tübingen. The study was conducted in accordance with the Declaration of Helsinki, and written informed consent was obtained from each patient. Only non-tumorous tissue was collected, as confirmed by histological examination, and stored at -80°C. Available patient documentation includes sex, age, smoking habits, alcohol consumption, presurgery medication, diagnosis leading to liver resection, and serological liver function parameters. Samples from patients with hepatitis, cirrhosis, or chronic alcohol abuse were excluded. A summary of the data is presented in Supplementary Table S1.

Phenotypic data were available from previous studies. Genome-wide mRNA expression profiling was previously performed using Illumina Human-WG6v2 Expression BeadChip (see below). For selected genes quantitative mRNA levels were determined by real-time PCR, protein levels by Western blot, and enzyme activity levels by mass spectrometry (Supplementary Table S2).

Genomic DNA was isolated from corresponding blood samples as described previously (Gomes et al., 2009). Quality and concentration of gDNA were determined using both, the Qubit Fluorometric Quantitation (Thermo Fisher Scientific, Dreieich, Germany) and Nanodrop ND-8000 (Thermo Fisher Scientific, Dreieich, Germany). Gene expression and genotyping data assessed by Human-WG6v2 Expression BeadChip and HumanHap300 Genotyping BeadChip (Illumina, Eindhoven, Netherlands) were preprocessed as previously described (Schröder et al., 2013) and the data are accessible through GEO Series accession numbers GSE32504 and GSE39036, respectively.

### Targeted ADME NGS Panel Sequencing

**FIGURE 1:**
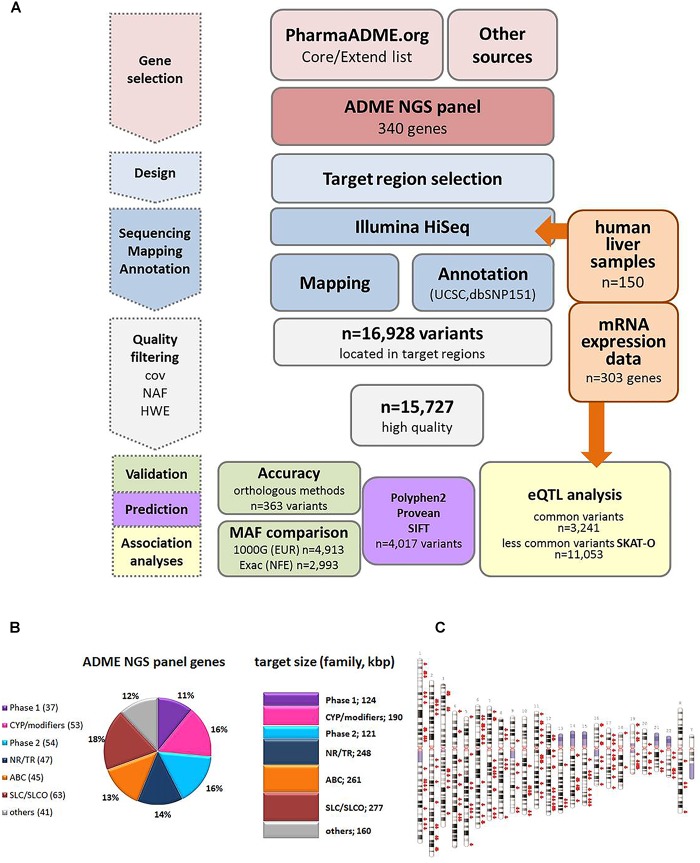
Study overview. **(A)** Schematic overview of the workflow for the ADME NGS panel sequencing. cov, coverage; NAF, novel allele frequency; HWE, Hardy–Weinberg equilibrium; MAF, minor allele frequency; eQTL, expression quantitative trait loci. **(B)** Composition of ADME NGS target genes displayed in % of total number (*n* = 340). Number of target genes within a family is given in brackets. Sum of target size is given in kbp. Major functional classes were defined as Phase 1, phase 1 enzymes; CYP/modifiers, cytochrome P450 and modifying enzymes; Phase 2, phase 2 enzymes; ABC, ABC transporters; SLC/SLCO, SLC/SLCO transporters and ion channels; NR/TR, nuclear receptors and transcriptional regulators; Others, other genes. For further details see Supplementary Table S1. **(C)** Ideogram of the genes included in ADME NGS panel. Target genes (*n* = 340) are denoted by red arrows besides chromosomes (GRCh37).

Genomic DNA was enriched using a custom design Agilent SureSelect XT in-solution kit (Agilent Technologies, Santa Clara, CA, United States). The design of the PGX panel for all relevant ADME classified and ADME related genes (340 genes in total) included publically available gene lists of PharmaADME.org^2^ (CORE/EXTEND, *n* = 236), pharmGKB^3^ (Whirl-Carrillo et al., 2012); [very important pharmacogenes (VIP), *n* = 36], as well as additional genes with confirmed or putative ADME-related function according to literature search (*n* = 104; Supplementary Table S2). For analysis, the genes were assorted into functional groups as follows: ATP-binding cassette transporters (ABC; *n* = 45), solute carrier transporters, solute carrier organic anion transporters, and ion channels (SLC/SLCO; *n* = 64), members of phase I metabolism excluding cytochrome P450 and other modifying enzymes (Phase1: *n* = 36), members of phase II metabolism (Phase 2; *n* = 53), cytochrome P450s/modifying enzymes (CYP/modifiers; *n* = 53), nuclear receptors/transcription regulators (NR/TR; *n* = 46), and genes of other background and potentially related to ADME (others; *n* = 43) (Figure 1B and Supplementary Table S2). Positions of exon regions, 3′ and 5′ UTR (untranslated regions) were based on RefSeq major transcripts sequences (GRCh37; hg19; UCSC genome browser). Exon sizes were extended by 20 nucleotides on each side. Sequence of very short exons was symmetrically increased to at least 160 nucleotides. For selected genes 5′ regions were extended to cover 2 kbp (*n* = 29). The total number of exons was 4,210 and total target size reached 1,382 kbp (Supplementary Table S2). Panel details are available on demand.

Target capturing was specifically designed for NGS of selected regions and DNA libraries were generated using Agilent in-solution target capture technology from up to 1 μg high quality genomic DNA for each sample. NGS was carried out on the Illumina HiSeq2500 system (Illumina Inc., San Diego, CA, United States) at high depth with 2 × 100 bps paired-end reads. Raw sequencing reads generated by the Illumina platform were demultiplexed using Illumina bcl2fastq (1.8.2) (Illumina, San Diego, CA, United States). Adapter sequences were removed with cutadapt and the trimmed reads mapped to the human reference genome (GRCh37 hg19) using the Burrows Wheeler Aligner (BWA-mem 0.7.2; Li and Durbin, 2010). Reads mapping to more than one location with identical mapping scores were discarded (in house software). Read duplicates likely resulting from PCR amplification were removed (samtools 0.1.18). Variants were called using samtools and varscan (2.3.5)^4^. Technical artifacts were removed (in-house software) and the remaining variants were annotated based on several internal and external databases. We created a read count matrix for sequenced targets and 150 samples using the R package cn.mops.1.12.0 and the BAM files to assess the quality of coverage per gene and per target region. Approximately 5.9 million on target reads were generated per sample with a mean mapping quality of 58.2 and a mean coverage of 343 per target site. A Frequentist or a Bayesian algorithm was applied to call SNVs and small insertions/deletions (INDELs). Detection of insertions is limited by read length and no insertions above 50 bp were observed. Variant annotations were retrieved from UCSC genome data browser^5^, dbSNP build151 (March 22, 2018), and Sequence Ontology (SO) terms to describe the effect of each variant on genes in terms of transcript structure. Enrichment and sequencing procedure were established, validated, and provided by CeGaT GmbH, Tübingen, Germany. CeGaT is accredited by DAkkS according to DIN EN ISO 15189:2014, by the College of American Pathologists (CAP) and CLIA-certified (Dohrn et al., 2017). Sequence variant data has been deposited at the European Genome-phenome Archive (EGA), which is hosted by the EBI and the CRG, under accession number EGAS00001003426. Further information about EGA can be found on https://ega-archive.org (Lappalainen et al., 2015).

### High Quality Variants

Only variants within the predefined target regions were selected and further analyzed (*n* = 16,928). Variant calls with sequencing coverage below 20× were regarded as invalid. Moreover, heterozygous calls were regarded as invalid when variant allele ratios were <5%. Invariant positions and variants with less than 70% valid values in all samples were excluded. Furthermore, 696 variants with HWE *p*-values < 10^-5^ were considered suspicious and consequently excluded from all subsequent analyses. Finally, 13,838 SNVs and 1,889 INDELs were further investigated in this work. Genedata Profiler Analyst Module (V12.0.2.; Genedata AG, Basel, Switzerland) and GraphPad Prism (V5.04; GraphPad Software Inc., La Jolla, CA, United States) were used for data filtering, visualization, and basic statistical calculations.

### Global Validation

Evaluation of ADME panel sequencing data was performed by direct comparison of sample genotypes to available genome wide SNP data (Illumina HumanHAP300 SNP; GEO Series accession number GSE39036; Schröder et al., 2013) as well as genotype data of 87 individual SNVs determinations obtained with several other genotyping methods in former studies (RFLP, Sanger sequencing, TaqMan allelic discrimination, MALDI-TOF, and other arrays) from the same sample set. Array variant data were “lifted” to GRCh37 (hg19), and only SNVs within the target regions defined above and with HWE *p*-value > 10^-5^ were extracted (*n* = 276). Finally, genotype data for 363 variants were available for validation. Concordance of genotype data from ADME NGS and results from orthogonal methods was evaluated by computing percentage of identical genotype calls over all variants and samples. Variant positions within the above defined target boundaries were extracted from publically available databases from the Exome Aggregation Consortium ExAC^6^ (Lek et al., 2016) and 1000 Genomes project^7^ (1000 Genomes Project Consortium et al., 2015). In total, 11,558 and 68,918 variants were retrieved in the demanded genomic regions from 1000G and ExAC, respectively. Chromosomal position and nucleotide change (reference/alternative) were used to identify corresponding variants in the ADME NGS panel data. After adjusting frequency data to MAF numbers ranging between 0 and 50%, MAF from European (EUR, 1000G) or non-Finnish European (NFE, ExAC) were compared to observed MAF from our cohort. In addition, several well known variants in *CYP2D6*, *CYP2C9*, *CYP2C19* and *CYP2B6, NAT2* and *DPYD* were confirmed by Sanger sequencing. A concordance of 100% was observed covering 57 SNVs in 19 samples.

### *In silico* Prediction

The impact of coding variants on protein function was predicted using Polyphen 2 (PP2^8^ Adzhubei et al., 2013) as well as the Provean Human Genome Variants tool [Protein Variation Effect Analyzer (PROV)^9^; Choi et al., 2012], providing Provean and in addition SIFT (Sorting Intolerant from Tolerant; Sim et al., 2012) scores. All algorithms are based, among other features, on sequence conservation and were used with default settings. For a total of 4,017 coding variants including missense (*n* = 3,893), frameshift (*n* = 37), initiator codon (*n* = 7), stop codon (*n* = 46) and other coding variants (Table 1), prediction was performed using chromosomal genomic positions, reference and variant nucleotide. Functional predictions of the type LOF versus tolerated (TOL) was retrieved from Provean (cutoff 2.5; deleterious/neutral), SIFT (cutoff 0.05; damaging/tolerated) and Polyphen2 (probably and possibly damaging/benign). It must be pointed out that frameshift variants (*n* = 37) as well as mutations of stop codons (gain/loss; *n* = 46) are not predictable by these tools.

**Table 1:** Structural classification of ADME panel variants (*n* = 15,727).

Coding (*n* = 6,058; 38.5%)			Non-coding (*n* = 9,669; 61.5%)		
Class^a^	Variant (known^b^)	Variant (novel^b^)	Class^a^	Variant (known^b^)	Variant (novel^b^)
Initiator_cod	3	4	Upstream	476	359
Missense	1,610	2,283	5′UTR	501	499
Stop_gained	22	22	Non-coding exon	95	34
Stop_lost	2		Intron	1,166	1,441
Synonymous	1,219	764	Splice	296	520
Inframe	29	28	3′UTR	2,922	1,261
Frameshift	19	18	Downstream	68	31
Other coding^c^	26	9			
**Total**	**2,930 (48%)**	**3138 (52%)**	**Total**	**5,524 (57%)**	**4,145 (43%)**


*^a^Classification nomenclature according to ENSEMBLE variation sequence ontology terms. ^b^Known/novel: with/without dbSNP database identifier. ^c^Including: coding-exon-variant, stop-retained*.

### *Cis*-eQTL Analyses

*Cis*-eQTL analysis between the 15,727 variants (13,838 SNVs and 1,889 INDELs) and their corresponding gene were performed with statistical software R-3.5.0 (R Core Team, 2018) and additional packages SNPassoc (v1.9-2; González et al., 2014), SKAT (v1.3.2.1; Lee, 2017), and illuminaHumanv2.db (v1.26.0; Dunning et al., 2015).

mRNA expression levels were assessed by Human-WG6v2 Expression BeadChip (Illumina, Eindhoven, Netherlands) and preprocessed as described (Schröder et al., 2013). Probe sets were re-annotated using the R package illuminaHumanv2.db (Dunning et al., 2015). Only probe sets with “good” or “perfect” probe quality as defined by illuminaHumanv2fullReannotation were considered for the eQTL analyses. Of the 340 ADME and ADME related genes described above, 303 genes (89%) were represented on the Human-WG6v2 Expression BeadChip with at least one “good” or “perfect” probe set. If several “good” or “perfect” probe sets were annotated to a gene, data of these entire probe sets (i.e., log2 normalized expression signals) were averaged, finally resulting in an expression matrix of size 303 genes × 150 samples for the eQTL analyses. Of the 15,727 variants, 14,294 (90.9%) were annotated to one of the 303 genes.

For individual eQTL analyses, only variants with MAF ≥ 2% and annotated to one of the 303 genes (*n* = 3,241) were considered, in order to avoid testing variants with very few minor allele carriers (a MAF ≥ 2% in 150 patients corresponds to at least 3 minor allele carriers; in our dataset, all variants with MAF ≥ 2% actually comprised at least 4 minor allele carriers). For 8 of the 303 genes, only variants with MAF < 2% were annotated in the ADME NGS panel (*ABCB9*, *ALDH2*, *CYP11A1*, *GSTK1*, *GSTM1*, *GSTT1*, *PRMT1*, and *SULT1A4*), leaving 295 genes and 3,241 variants for individual *cis*-eQTL analyses. These analyses were performed using the generalized linear model framework of R-package SNPassoc (González et al., 2014), considering four different genetic models: codominant, dominant, recessive, and additive. Only the minimal *p*-value of the four genetic models for each SNP was reported. Besides univariate analyses, *cis*-effects of variants on mRNA expression were analyzed controlling for 10 covariates [sex, age, smoking, alcohol consumption, diagnosis, C-reactive protein (CRP) level, cholestatic liver disease, presurgical medication (no drugs, P450 inducer and other drugs), serum total bilirubin (TBILI) level, and serum gamma glutamyl transferase (GGT) level; see further details in Supplementary Table S1]. We used the Bonferroni method for multiple testing correction and set the significance level at 0.05/3,241 = 1.54E-05.

Moreover, we performed combined *cis*-eQTL analyses of the rare variants (MAF < 2%; *n* = 11,053) using the optimal unified association test framework for sets of variants (SKAT-O; Lee et al., 2012) implemented in R-package SKAT. To be more precise, for each of the 303 genes, the association of the set of all rare variants annotated to this gene and the corresponding mRNA expression data was investigated applying the SKAT-O test with standard weights. The same 10 covariates as in the eQTL analysis of common variants were used for an analogous multivariate SKAT-O analysis. For combined *cis*-eQTL analysis of rare variants, the Bonferroni-corrected significance level was set to 0.05/303 = 1.65E-04.

## Results

### Development and Performance of the Targeted ADME NGS Panel

Figure 1A gives an overview of the project workflow. The selection of genes was based on the PharmaADME.org gene lists “core” and “extend” and the PharmGKB VIP genes and was complemented with numerous additional genes of potential relation to drug metabolism (Figure 1B). All 340 genes finally included were targeted for all exons, exon/intron boundaries, as well as 5′ and 3′UTRs. An extended 5′ region of 2 kb was included for a group of 29 selected genes. The total panel size comprised 1,382 kbp distributed over all chromosomes except the Y chromosome (Figure 1C and Supplementary Table S2). In our cohort of 150 liver samples, the gene target regions were covered to a mean read-depth of 343× (25th percentile = 265; 75th percentile = 398; Supplementary Figure S1A). More than 98% of the target regions were covered at more than 30×. The highest coverage was obtained for *UGT2B11* (average 811), while *GSTT2B* showed the lowest average coverage of 27. These discrepancies did not hinder our analysis and can be resolved in a further iteration of design. Overall, 99% of the genes were covered on average at least 100-fold. Direct comparison of variant annotation with 363 available genotypes determined independently by other methods revealed an overall concordance of >99% (Supplementary Figure S2). The accuracy obtained with data derived from the Illumina HumanHap300 genotyping platform (99.3%) was slightly lower compared to data from other genotyping methods (99.6%), which may be due to inaccurate genotype calling by the array method. Further details on performance and validation of the ADME NGS panel are presented in the Sections “Materials and Methods” and Supplementary Material.

### Analysis of DNA Variants

A total of 16,928 genetic variants were detected within the defined target regions. Of these, 1,201 were excluded from further analysis because of low genotype quality (*n* = 505) or due to HWE *p*-values below 10^-5^ (*n* = 696). The remaining 15,727 variants comprised 13,838 SNV and 1,889 variants classified as small insertions or deletions (INDELs). The length changes of these ranged from deletion of 33 nucleotides up to insertion of 20 nucleotides, with 1 bp deletions or insertions being the most frequent. Larger structural variants including copy number variations (CNVs) are currently under investigation using other methods.

As expected, most SNVs were biallelic, only 62 were triallelic and no tetraallelic variants were found. Among triallelic variants, transversions were more common (*n* = 80) than transitions, and G to T and G to A were the most common observations (*n* = 26 and *n* = 25, respectively).

None of the sequenced regions was invariant. On average, we observed 10.5 variants/kbp, corresponding to a mean distance of variants of 95 bp. Based on SNV density, the least variable genes were *UGT1A9* and *UGT1A10* with <2 SNVs/kbp and the genes with highest observed variant densities were *CYP4F11* (42 SNVs/kbp) and *CYP2D6* (31 SNVs/kbp) (Supplementary Figure S1C).

**FIGURE 2:**
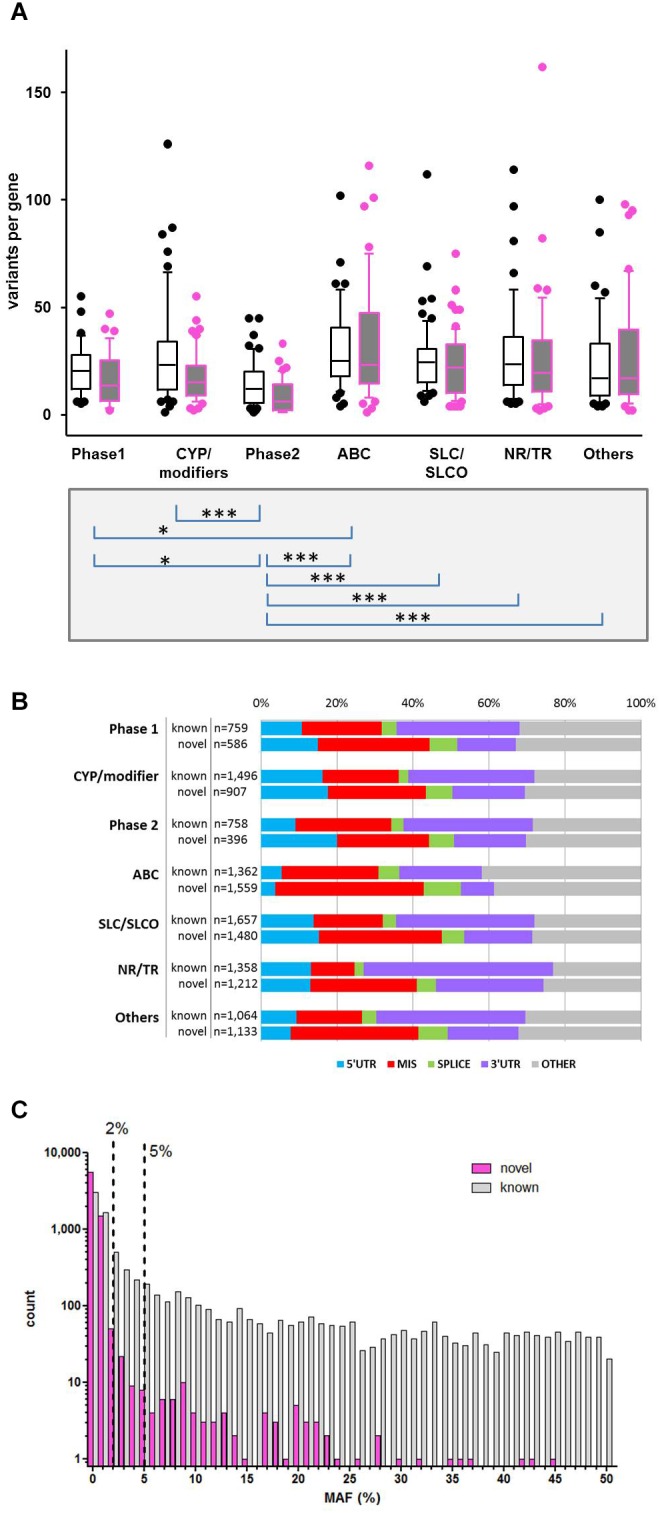
Variability of gene families. **(A)** Distribution of known and novel variants in ADME gene families. The numbers of observed known and novel variants (including SNVs and INDELs) per gene are shown for the seven major functional classes of ADME genes defined in Figure 1. Open boxes, known variants; filled boxes, novel variants; boxes show median with 75th and 25th percentiles and whiskers represent 10th and 90th percentiles. Lower part: statistical significance calculated by Kruskal–Wallis with Dunn’s multiple comparison test of total number of variants per genes between family groups: ^∗^*P* ≤ 0.05, ^∗∗∗^*P* ≤ 0.001. **(B)** Functional categorization of variants. Total number and proportion of variants observed in each functional class is shown separately for known and novel variants. Functional classes are defined as follows: 5′UTR, upstream and 5′ untranslated region; MIS, initiator codon, missense and stop codon variants; SPLICE, variants in consensus splice site acceptor and donor regions; 3′UTR, downstream and 3′ untranslated region; OTHER, other functional classes (intronic, frameshift, synonymous, other coding and non-coding variants). **(C)** Comparison of minor allele frequencies (MAF) between novel and known observations. Total number of known observations with dbSNP identifier (open white bars; *n* = 8,454), novel observations (filled purple bars; *n* = 7,273); dotted line marks MAF = 2 and 5%.

Variant annotation revealed that 7,273 (46.2%) of the variants were not yet annotated in the NCBI dbSNP database (dbSNP build 151, March 2018) and thus considered as novel observations. Figure 2A displays the number of variants per gene for known and unknown variants in the different ADME gene groups while Figure 2B depicts the fraction of variants according to functional annotation. The number of variants per gene was highest in the ABC and SLC/SLCO transporters and lowest in phase II genes. As reported in several recent studies the number of novel observations was substantial in all gene and functional groups (Fujikura et al., 2015; Gordon et al., 2016; Han et al., 2016). Of 15,727 SNV and small INDEL variants, 12,022 had a MAF below 2%, including 8,937 singletons. Of the 7,273 novel variants, 7,139 (>98%) had MAFs below 2% (Figure 2C), while 80 (1.1%) had MAFs ≥ 5%. Most of these were located in non-coding regions.

Functional classification based on major transcripts for each gene according to UCSC database revealed 6,058 variants in coding regions (including 3,893 missense and 46 stop gain variants; Table 1 and Figure 2B) and 9,669 variants in various non-coding regions (e.g., 1,000 in 5′UTR and 4,138 in 3′UTR; Table 1 and Figure 2B). We also analyzed 36 VIP genes, derived from PharmaGKB and PharmaADME websites separately for novel SNVs. In total we observed 502 unannotated variants in these genes (dbSNP151), 120 of them representing missense variants (Supplementary Table S3).

For comparison with publically available population data, we extracted small variants from the 1000 Genomes (EUR population) and ExAC (NFE, non-Finnish European) databases for the ADME NGS panel target regions, resulting in 11,558 and 68,918 variants, respectively (Supplementary Figure S3A). The MAFs of the matching variants in our sample set (ExAC/NFE: *n* = 2,993; 1000G/EUR: *n* = 4,913) were in good correlation with published population frequency data (Pearson *r* = 0.96 and *r* = 0.98 for both EUR and NFE populations, respectively). The median MAF of these SNVs was 1.16% for NFE and 2.98% for EUR. We did not detect another 6,645 (EUR) and 65,925 (NFE) known variants with median MAFs of 0.1% (EUR) and 0.002% (NFE) (Supplementary Figures S3A,B). Together these data indicate that mainly very rare variants with allele frequencies below 0.1% were missed in our cohort.

### Association With Expression Levels

To directly evaluate the functional impact of variants, we assessed liver mRNA expression in an existing dataset (Schröder et al., 2013). To ensure high data quality only mRNA expression data of genes with “perfect” or “good” probes (see section “Materials and Methods”) were considered (available for *n* = 303 genes). Due to sample size and statistical power considerations, we performed separate analyses for less common (MAF < 2%) and more common (MAF ≥ 2%) variants.

**FIGURE 3:**
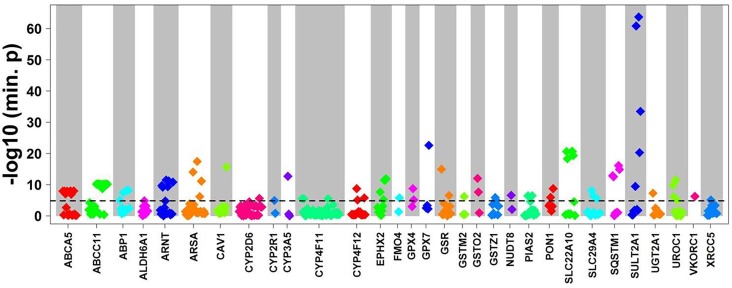
*Cis*-eQTL analysis of common variants. Top *cis*-associations of common variants to mRNA expression. Manhattan plot presenting top results from multivariate cis association analysis between mRNA expression and common variants (MAF ≥ 2%) investigated for the corresponding gene. Displayed are minimal *p*-values (min. *p*) from four genetic models (codominant, dominant, recessive, and additive). In total, *n* = 3,241 common variants in *n* = 295 genes were analyzed. Only genes with at least one significant *cis*-association after Bonferroni correction (*p* < 0.05/3,241 = 1.54E-05; dotted line) are shown with all minimal *p*-values. The significant *p*-values are presented in Table 2.

To evaluate the impact of more common variants (*n* = 3,241) on expression of the corresponding genes we performed *cis*-eQTL analysis using univariate regression models. This analysis revealed significant associations for 94 variants after Bonferroni correction. In multivariate analysis with correction for 10 covariates (see section “Materials and Methods”) 90 variants in 31 genes remained significant after Bonferroni correction (minimal *p*-value of the four genetic models < 1.54E-05; Figure 3 and Table 2). Interestingly, 62 (70%) of these were located in non-coding regions, and most of these (*n* = 40) in 3′UTR regions. Of note, three eQTLs represented PharmGKP VIP genes (CYP2D6: rs1080985; CYP3A5: rs15524; VCORC1: rs7294).

**Table 2:** eQTL analysis: Significant associations from multivariate regression models after Bonferroni correction (only minimal *p*-values of four genetic models used are listed).

Gene	Variant^a^	dbSNP151	Functional class	Minimal *p*-value^b^
*ABCA5*	17_67242245_G_A	rs12942867	3′UTR	1.20E-08 A
	17_67242551_AG_A	rs321469	3′UTR	1.20E-08 A
	17_67242756_G_A	rs1990248	3′UTR	9.00E-08 A
	17_67243289_A_T	rs15886	3′UTR	1.20E-08 A
	17_67260926_A_G	rs12449649	Synonymous	1.00E-08 A
	17_67267317_T_C	rs557491	Missense	1.10E-07 A
	17_67282332_T_C	rs1550828	Intron	1.00E-08 A
*ABCC11*	16_48250011_G_A	rs11863233	Intron	6.90E-11 A
	16_48250026_G_T	rs11863236	Missense	6.90E-11 A
	16_48250218_T_C	rs28654935	Intron	6.90E-11 A
	16_48256602_T_C	rs16945974	Synonymous	6.90E-11 A
	16_48265777_C_T	rs16945988	Missense	6.90E-11 A
	16_48269120_TAGAGATGCAA_T	rs398088092	Upstream	3.20E-10 C
	16_48269140_AAGAGATGCAA_A		Upstream	1.80E-09 A
	16_48269561_A_G	rs10521167	Upstream	6.90E-11 A
	16_48269918_T_C	rs16946006	Upstream	6.90E-11 A
	16_48270429_C_T	rs9926206	Upstream	6.90E-11 A
	16_48270508_T_C	rs9934833	Upstream	6.90E-11 A
	16_48270574_A_G	rs9932328	Upstream	6.90E-11 A
*AOC1*	7_150553605_C_T	rs10156191	Missense	8.50E-06 A
	7_150555915_A_G	rs10893	Synonymous	3.40E-08 R
	7_150557622_G_A	rs12179	Synonymous	6.50E-09 R
	7_150557665_C_G	rs1049793	Missense	6.50E-09 R
	7_150558366_C_T	rs12539	3′UTR	6.30E-09 D
*ALDH6A1*	14_74527190_A_G	rs8204	3′UTR	1.40E-05 C
*ARNT*	1_150783934_G_GCACA	rs71580328	3′UTR	1.90E-10 D
	1_150783934_G_GCACACA	rs71580328	3′UTR	5.80E-10 C
	1_150783985_T_C	rs11552229	3′UTR	3.10E-12 A
	1_150804401_G_GA	rs200891935	Intron	4.60E-10 C
	1_150808889_C_G	rs2228099	Synonymous	6.70E-12 A
	1_150850904_CA_C	rs10305645	Upstream	1.50E-11 D
*ARSA*	22_51062832_G_A	rs8142033	3′UTR	9.10E-15 A
	22_51063477_T_C	rs6151429	3′UTR	3.70E-18 A
	22_51064039_G_C	rs743616	Missense	6.40E-07 A
	22_51064416_T_C	rs2071421	Missense	7.30E-12 A
*CAV1*	7_116200587_C_T	rs1049337	3′UTR	2.50E-16 A
*CYP2D6*	22_42528382_C_G	rs1080985	Upstream	2.40E-06 D
*CYP2R1*	11_14900931_G_A	rs117913124	Synonymous	1.10E-05 D
*CYP3A5*	7_99245914_A_G	rs15524	3′UTR	2.10E-13 D
*CYP4F11*	19_16023318_C_G	rs61175303	3′UTR	3.10E-06 C
	19_16023378_G_A	rs58046343	3′UTR	3.10E-06 C
	19_16023619_T_C	rs58153611	3′UTR	3.10E-06 C
*CYP4F12*	19_15791132_T_A	rs2074568	Intron	1.80E-09 A
	19_15793235_T_C	rs2285888	Missense	8.30E-06 A
	19_15807884_A_G	rs593818	Missense	1.80E-06 A
*EPHX2*	8_27373923_T_C	rs4149243	Splice_region	2.20E-08 D
	8_27396208_G_A	rs4149253	Synonymous	5.80E-06 C
	8_27401964_A_C	rs1126452	Synonymous	3.60E-12 D
	8_27402074_A_G	rs1042032	3′UTR	3.60E-12 D
	8_27402132_T_C	rs1042064	3′UTR	1.20E-12 D
*FMO4*	1_171311003_A_C	rs1042772	3′UTR	1.70E-06 A
*GPX4*	19_1106477_G_C	rs8178977	Intron	1.80E-09 A
	19_1106615_T_C	rs713041	3′UTR	6.90E-06 A
*GPX7*	1_53074532_C_A	rs1047635	3′UTR	2.50E-23 C
*GSR*	8_30535660_C_A	rs3594	3′UTR	1.10E-15 A
	8_30536581_A_G	rs1138054	3′UTR	2.90E-07 A
*GSTM2*	1_110210780_C_G	rs530021	Splice_region	6.10E-07 A
*GSTO2*	10_106034491_A_G	rs2297235	5′UTR	1.00E-12 A
	10_106037894_T_C	rs157077	Intron	2.40E-08 A
*GSTZ1*	14_77788908_G_A	rs2363643	Intron	1.40E-06 A
	14_77793207_G_A	rs7975	Missense	8.70E-06 A
*NUDT8*	11_67395714_C_T	rs7124513	Synonymous	2.50E-07 A
*PIAS2*	18_44390536_T_C	rs17472	3′UTR	3.50E-07 A
	18_44391566_T_TAG	rs149022619	3′UTR	3.50E-07 A
*PON1*	7_94927924_C_T	rs854552	3′UTR	1.20E-06 A
	7_94953895_G_A	rs705379	Upstream	1.90E-09 A
*SLC22A10*	11_63057925_G_A	rs1790218	Stop_gained	2.50E-21 C
	11_63064823_T_C	rs576641	Synonymous	5.10E-19 A
	11_63072310_C_T	rs1201559	Missense	2.50E-21 C
	11_63078986_T_C	rs1404608	3′UTR	2.50E-21 C
	11_63079101_AT_A	rs5792282	3′UTR	4.40E-20 A
*SLC29A4*	7_5338714_T_C	rs6950111	Synonymous	8.20E-09 A
	7_5342413_T_C	rs11979775	Intron	4.10E-07 D
	7_5342980_C_T	rs56166050	3′UTR	2.80E-06 D
*SQSTM1*	5_179260153_C_T	rs4935	Synonymous	1.40E-13 A
	5_179260213_G_A	rs4797	Synonymous	2.80E-13 A
	5_179264731_T_C	rs10277	3′UTR	7.60E-17 A
	5_179264915_G_T	rs1065154	3′UTR	1.30E-15 A
*SULT2A1*	19_48374306_G_A	rs112468411	3′UTR	3.50E-10 C
	19_48374320_C_T	rs112285002	3′UTR	1.50E-61 C
	19_48374538_T_C	rs296366	3′UTR	1.90E-64 C
	19_48374551_C_G	rs296365	3′UTR	5.60E-21 R
	19_48389363_G_A	rs296361	Intron	3.30E-34 C
*UGT2A1*	4_70454289_A_G	rs4148312	3′UTR	5.20E-08 A
*UROC1*	3_126200146_A_T	rs777513	3′UTR	1.40E-10 A
	3_126200291_C_T	rs800950	3′UTR	7.70E-07 R
	3_126200403_A_C	rs1799398	3′UTR	3.10E-12 A
	3_126202257_G_A	rs1687477	Synonymous	8.50E-06 R
*VKORC1*	16_31102321_C_T	rs7294	3′UTR	5.50E-07 A
*XRCC5*	2_217012901_A_G	rs207906	Synonymous	7.60E-06 D


*^*a*^Variant identifier “chromosome _ position _ reference nucleotide _ variant nucleotide”.^*b*^Genetic model with minimal p-value: A, additive; R, recessive; D, dominant; C, codominant*.

**FIGURE 4:**
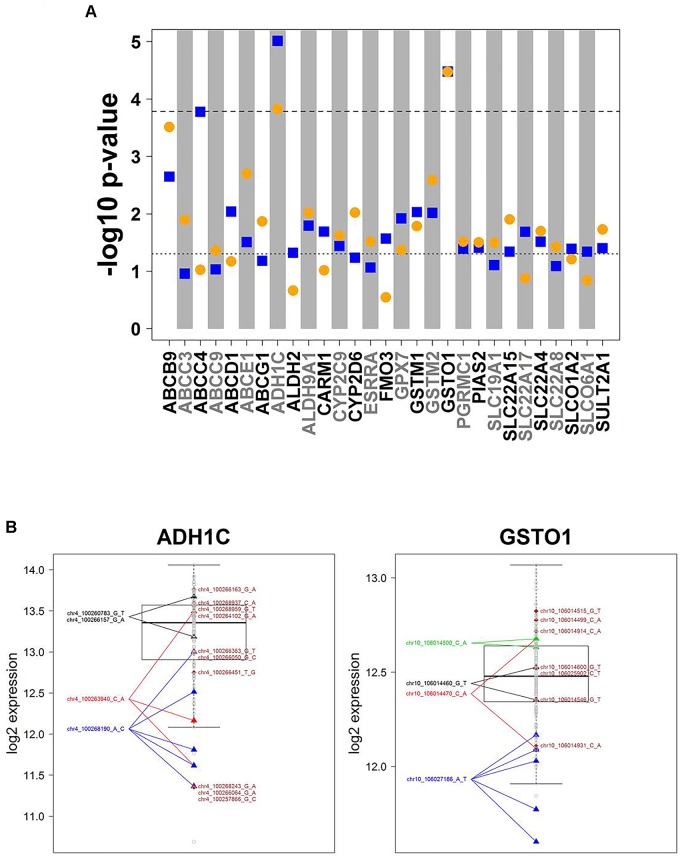
*Cis*-associations of rare variants and mRNA expression (SKAT-O analysis). **(A)** Manhattan plot displaying SKAT-O test *p*-values from uni- and multivariate *cis*-association analysis between mRNA expression and the set of all rare variants (MAF < 2%) investigated for the corresponding gene. In total, *n* = 11,053 rare variants in 303 genes were analyzed. Only genes with a minimal association *p*-value < 0.05 are shown. Horizontal dotted lines indicate significance level at 0.05 (lower) and Bonferroni corrected significance level at 0.05/303 = 1.65E-04 (upper). Blue squares: univariate analysis, orange circles: multivariate analysis. **(B)** Boxplots of ADH1C and GSTO1 gene expression, the two genes with SKAT-O test *p*-values < 1.65E-05 in both uni- and multivariate analysis. All variants are heterozygous. Patients with rare variants (MAF < 2%) in *ADH1C* or *GSTO1* are marked by triangles if several patients are carrying a rare mutation or diamonds if a rare mutation is only present in one patient. Gray dots represent patients without rare variants for the gene displayed. Colors differentiate variants.

Association analysis of rare variants is challenging. To overcome the problem of limited sample size/statistical power, various methods have been developed to test sets of rare variants. Here we used the SKAT-O approach (Lee et al., 2012) for group-wise association of all rare variants in a gene with mRNA expression data. These variants are incorporated into a gene-wise test statistic via a weighted sum. Thus, *p*-values relate to genes, not to variants. SKAT-O combines the strengths of burden tests thereby being powerful in different scenarios, i.e., when many variants of a gene are associated with expression levels and have the same effect direction, or when there are only few associated variants or variants that differ in effect direction. Figure 4A summarizes the results for univariate and multivariate SKAT-O analyses. After correction for multiple testing, two associations, for *ADH1C* and *GSTO1*, remained statistically significant. Further details showing expression levels of individual carriers are presented in Figure 4B. For example, five samples with a rather low expression were heterozygous carriers of the SNP chr10_106027186 A > T (3′UTR; rs17885600), including the two individuals with the lowest GSTO1 levels (Figure 4B). Hence, SKAT-O analysis resulted in identification of at least two genes with plausible genotype–phenotype correlations for variants with MAF < 2%.

### Prediction of Functional Effects

**FIGURE 5:**
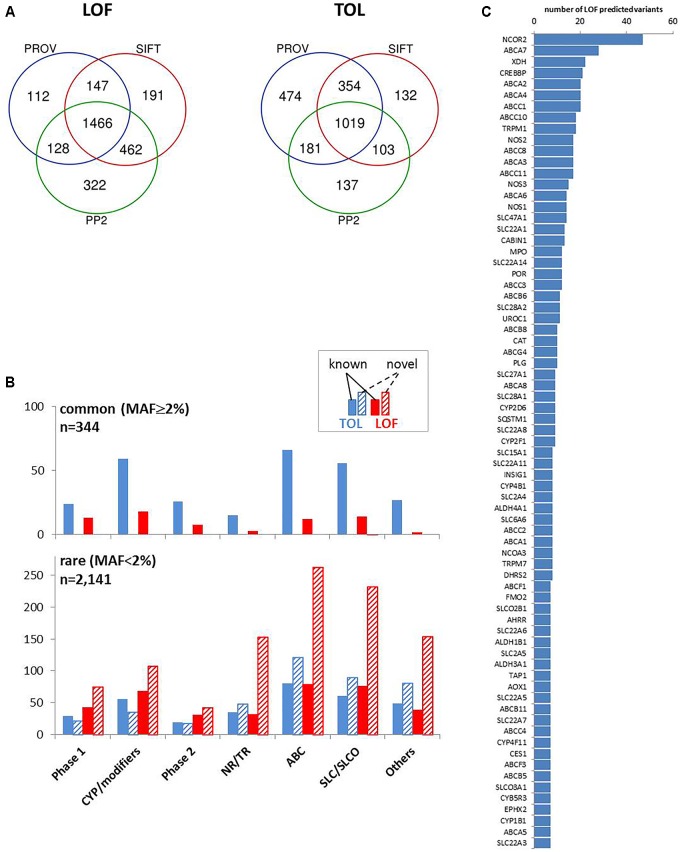
Prediction of coding variant effects. **(A)** Comparison of loss-of-function (LOF) and tolerable (TOL) predictions obtained by three different prediction tools. Venn diagrams are shown for “LOF” and “TOL” predictions for *n* = 4,017 coding variants from Provean (“deleterious”), SIFT (“damaging”), Polyphen2 (PP2; “probably/possibly damaging”). **(B)** Occurrence of TOL and LOF variants in gene family groups. The distribution of the number of concordant TOL (*n* = 1,019; blue colored) and LOF (*n* = 1,466; red colored) predictions is shown for the indicated gene groups for known (filled bars) and novel (hatched bars) variants. Upper chart: variants with MAF ≥ 2%; lower chart: variants with MAF < 2%. **(C)** Top LOF-variant carrier genes. Shown are genes with at least seven predicted LOF-variants.

We concentrated on coding variants resulting in amino acid change (missense), frameshift, or affecting initiator and stop codons, together accounting for 66% of coding variants and one fourth of all variants (Figure 2B). We used the common tools Polyphen 2 (PP2), Provean, and SIFT, that make dichotomous functional predictions of the type “loss of function” (LOF) versus “tolerated” (TOL) (Zhou et al., 2018a). Of the analyzed subset of 4,017 coding variants, more than 95% were predictable by these algorithms (PP2, *n* = 3,818; PROV, *n* = 3,874; SIFT, *n* = 3,881). LOF prediction was retrieved concordantly by all three algorithms for 1,466 variants (36.5%) and TOL was concordantly calculated for 1,019 variants (25.4%; Figure 5A). In agreement with other studies (Bush et al., 2016; Han et al., 2016; Hovelson et al., 2017) we found that the proportion of LOF- versus TOL-predicted variants was significantly higher among the less common (MAF < 2%) compared to more common variants (Chi-square test, *p* < 0.0001). With one exception (*SLC28A1* G254V, MAF = 2.3%) all novel LOF-predicted variants were less common with MAF < 2% (Figure 5B).

Interestingly, transporters and nuclear receptors/transcriptional regulators had large proportions of predicted LOF variants that had not yet been listed in the dbSNP database. The highest number of predicted LOF variants in one gene was observed in *NCOR2* (*n* = 47), and nine ABC transporters (*A7*, *A2*, *A4*, *C1*, *C10*, *C8*, *A3*, and *C11*) are found among the genes with the highest LOF-predicted variants (Figure 5C).

### Integrating Prediction and Association

**FIGURE 6:**
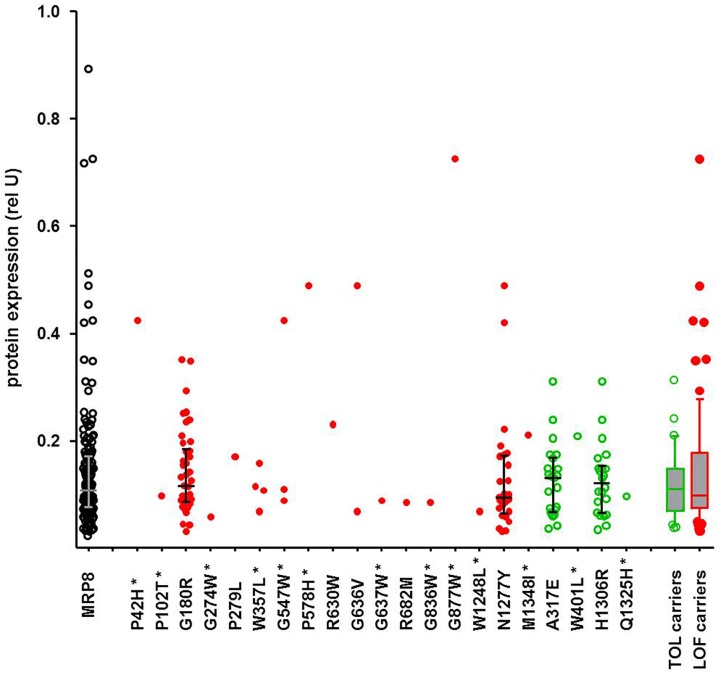
Genotype-phenotype relation of *ABCC11* missense variants to MRP8 protein expression. Relative MRP8 protein abundance in the same human liver samples used for NGS was determined by Western blot analysis (Magdy et al., 2013). Symbols: open black circles, all variants; red filled circles, carriers of LOF-predicted variants; green open circles, carriers of only TOL-predicted variants; green box and whisker: carriers of TOL-predicted variants not carrying LOF-variants (*n* = 30); red box and whisker: carriers of at least one LOF (*n* = 73). Novel variants are indicated by a star.

While the SKAT-O test identified only two significant associations, functional prediction indicated a much larger number of predicted LOF variants, as also reported by others (Han et al., 2016; Hovelson et al., 2017). In contrast to former studies, our data allow inspection of genotype-phenotype correlations individually for each variant and for several available phenotypes. While these excessive data are currently being analyzed, we illustrate here a typical example. Of particular interest are protein levels, as functionally damaging ADME gene variants are frequently associated with lower protein levels. Figure 6 shows exemplarily the correlation of all detected amino acid variants of *ABCC11*, encoding the drug transporter MRP8, with MRP8 protein levels obtained for the same liver cohort in a previous study (Magdy et al., 2013). Interestingly, carriers of concordantly LOF-predicted variants (*n* = 73) showed highly variable protein levels (23-fold; coefficient of variation 81%), essentially covering the entire range of MRP8 variability, while carriers of only TOL-predicted variants (*n* = 30) were spread across a smaller protein range (ninefold; coefficient of variation 53%). Of note, the median protein levels of carriers of LOF-predicted and TOL-only-predicted variants were similar (*P* = 0.73; Figure 6). Thus, our phenotypic data allow identification of several MRP8 low and high expressors in relation to genotype. While there does not seem to be a simple relation between functional prediction and phenotypic expression, our data should be helpful to prioritize variants for further investigation and to improve prediction tools.

## Discussion

In this study we designed a new panel to target 340 ADME genes for NGS. We tested and validated our ADME NGS panel on a cohort of 150 human liver specimens with comprehensive genetic, functional, and medical characterization. This allowed us not only to perform extensive genotype-phenotype correlations to identify novel relationships for common and rare variants but also to compare computational predictions of functional effects with real phenotypes, which should be useful to further develop and optimize prediction algorithms for variant effects.

We designed our ADME NGS panel to comprise 340 genes including most phase I and phase II enzymes, drug transporters and numerous transcriptional regulators and other modifiers of xenobiotics and endogenous substances. We used Agilent in-solution target capture technology to allow informed selection of relevant regions and optimization of coverage on targets. Only four genes, *SULT1A3*, *SULT1A4*, *MIF*, and *CYP26C1*, were covered below 100-fold. Low coverage of some genes was also observed by others who speculated that common null functional alleles, high sequence homology as well as pseudogenes may disturb capture of such regions (Han et al., 2016). Direct comparison of 363 genotype data available from previous pharmacogenetic studies in the liver cohort revealed an overall accuracy of the ADME NGS panel of >99%. The overall performance of our ADME NGS panel was comparable to other targeted capture sequencing panels (Bush et al., 2016; Gordon et al., 2016; Han et al., 2016; Hovelson et al., 2017). Compared to these other platforms we included a greater number of genes with the intention to investigate not only established ADME genes but also less well known ADME candidate genes.

While several NGS studies of different types recently explored genetic variation in ADME genes (Fujikura et al., 2015; Bush et al., 2016; Han et al., 2016; Kozyra et al., 2016; Hovelson et al., 2017; Schärfe et al., 2017), our study is, to our knowledge, the only one that provides phenotypic measurements in human samples. In this study we analyzed only SNVs and small INDELs, while larger structural variations will be analyzed separately (Tremmel et al., in preparation). For the more common variants (MAF ≥ 2%) multivariate eQTL analysis revealed 90 significantly associated variants, most of them located in non-coding regions. Six of these loci had already been described in our previous genome wide association study, e.g., rs7294 in *VKORC1* 3′UTR, or rs1201559 (P516L) in *SLC22A10* (Schröder et al., 2013). Interestingly, several of the SNVs located in 3′UTRs (*ARNT* rs11552229, *CYP3A5^∗^10* rs15524, *EPHX2* rs1042032 and rs1042064, *UGT2A1* rs4148312 and *VKORC1* rs7294) are discussed as potential micro-RNA binding sites, partially proven by tissue eQTL (Wei et al., 2012). Furthermore, our data confirm predicted eQTL effects on expression in liver tissue in the Genotype-Tissue Expression portal (GTex^10^; Lonsdale et al., 2013) e.g., for the *EPHX2* variant rs1042032 and *VKORC1* rs7294. Some other eQTLs we found had also been reported previously in the context of phenotype/genotype correlations. For example, rs1080985 in *CYP2D6* corresponds to the -1584C > G variant that is linked to the low-expression *CYP2D6^∗^41* allele (Raimundo et al., 2000; Raimundo et al., 2004); the *PON1* rs854552 variant had been found in a nutrigenetic approach on markers of cardiovascular disease (Rizzi et al., 2016); and the *AOC1* (diamine oxidase) variant rs10156191 was associated with hypersensitivity response to non-steroidal anti-inflammatory drugs (Agúndez et al., 2012).

In contrast to common variants, association of individual rare variants is greatly limited by sample size and thus presents a special challenge. The problem is aggravated by the fact that by far most rare variants occur in heterozygous condition, where any effect could be masked by the variability of the “normal” allele. Furthermore, rare variants can be damaging in many ways, affecting expression, protein abundance, or catalytic function. A single phenotype such as expression may thus not reveal the deleterious nature of a particular variant. Nevertheless we assume that analysis of gene or protein expression should be most promising, because damaging variants often affect expression negatively. This is the case, for example, for most low-activity CYP variants (e.g., CYPs 2B6, 2C19, 2D6, 3A4, 3A5 mostly due to aberrant splicing; Zanger and Schwab, 2013), and many established variants of clinical relevance like *UGT1A1^∗^28* and Gilberts syndrome (Ehmer et al., 2012) and *VKORC1* variants in warfarin metabolism (Li et al., 2009). Our statistical approach to relate rare variants to gene expression data by SKAT-O test revealed two significant associations for rare variants of *ADH1C* and *GSTO1*, both of which appear highly plausible and would not have been detected by the *cis*-eQTL analysis. The variant rs283413 in *ADH1C*, a stop gain mutation at protein position G78, is discussed as risk factor for Parkinson’s disease (Buervenich et al., 2005) and alcohol biodisposition (Martínez et al., 2010; Way et al., 2015). The *GSTO1* rare variants have so far not been reported to be associated with expression to our knowledge, but a significant genotype influence of the 3′UTR SNP rs17885600 on expression of the adjacent *GSTO2* in liver tissue supports a potential eQTL effect of this variant (Lonsdale et al., 2013).

As a further approach to identify deleterious ADME rare variants, we used computational prediction, which has recently been used in several studies (Bush et al., 2016; Han et al., 2016; Hovelson et al., 2017). However, in none of these studies, phenotypic information was provided to compare prediction with a phenotypic parameter. Similar to other studies we found a considerable fraction of all variants (36.5%) to be predicted as damaging by all three prediction tools used. Somewhat unexpectedly, preliminary analyses did not reveal statistically significant associations between LOF-predicted variants and lower expression. As exemplarily illustrated for *ABCC11* and MRP8 protein abundance, LOF predicted variants were not more frequently associated with lower protein levels as compared to TOL predicted variants. Thorough analyses of these data are currently in progress. A recent advanced approach integrated prediction and functional activity data available from diverse sources to develop an improved prediction framework adopted to pharmacogenetic assessments (Zhou et al., 2018b). Our data should be highly valuable to test and further improve such approaches.

## Conclusion

We designed a new targeted NGS pipeline to determine SNVs and small INDELs for 340 ADME genes and used it to analyze 150 well characterized human liver samples. In addition to common known variants we confirmed the existence of large numbers of rare and previously unknown germline variants. Available phenotypic information on the samples allowed us to elucidate numerous novel eQTLs for common variants and to identify novel relationships between rare variants and expression. Furthermore our data allow direct comparison of computationally predicted functional effects for coding variants with actual phenotypes. Using data for the transporter *ABCC11*/MRP8, we showed that variants predicted as deleterious are present in both high and low expressors of MRP8. While this emphasizes challenges and current limitations of computational prediction approaches to integrate rare variants into pharmacogenomics, such data are important to assess and improve the current strategies.

## Author Contributions

KK, ES, MS, UZ, SF, and SB designed the study. KK, UZ, and MS provided DNA samples. SF, FB, TS, and SB designed the panel and generated sequencing data. KK, RT, SW, and SF analyzed the data. KK, RT, and UZ wrote the manuscript. All authors contributed to editing and final proofreading the manuscript.

## Conflict of Interest Statement

SF, FB, TS, and SB were employed by CeGaT GmbH, Tübingen. The remaining authors declare that the research was conducted in the absence of any commercial or financial relationships that could be construed as a potential conflict of interest.

## Footnotes

1 www.pharmgkb.org/guidelines

2 http://pharmaadme.org

3 https://www.pharmgkb.org/

4 http://dkoboldt.github.io/varscan

5 https://genome.ucsc.edu/cgi-bin/hgVai

6 http://exac.broadinstitute.org/

7 http://www.internationalgenome.org/

8 http://genetics.bwh.harvard.edu/pph2/

9 http://provean.jcvi.org/index.php

10 https://commonfund.nih.gov/gtex

## Abbreviations

ADME: Absorption Distribution Metabolism Excretion
bp: basepair
CNV: copy number variant
eQTL: expression quantitative trait loci
HWE: Hardy–Weinberg equilibrium
INDEL: insertion/deletion
Kbp: kilo basepair
LOF: loss of function
MAF: minor allele frequency
NGS: next generation sequencing
RFLP: restriction fragment length polymorphism
SNP: single nucleotide polymorphism
SNV: single nucleotide variant
TOL: tolerated
UTR: untranslated region
