# Supplementary material for: A New Panel-Based Next-Generation Sequencing Method for ADME Genes Reveals Novel Associations of Common and Rare Variants With Expression in a Human Liver Cohort
Source: Front Genet. 2019 Jan 31;10:7. doi: 10.3389/fgene.2019.00007 (PMC6365429; doi:10.3389/fgene.2019.00007)
Supplement: Supplementary file 1 [file Table_1.DOCX]

**Supplement Table 1.** Population demographics and clinical/pathophysiological data of liver donors.

| **Subgroup** | **Categories** | **Number** |
| --- | --- | --- |
| Sex | Male | 71 |
|  | Female | 79 |
|  |  |  |
| Age | median [range]: 59 [7-85] | |
|  |  | |
| Smoking habitus^a^ | non-smoker | 117 |
|  | smoker | 29 |
|  |  |  |
| Alcohol consumption^a^ | none | 96 |
|  | ≥1 times/week | 48 |
|  |  |  |
| Presurgery drug exposure | none | 40 |
|  | P450-inducers^b^ and/or | 110 |
|  | other drugs |  |
|  |  |  |
| tBili (mg/dL)^a^ | normal (≤1.2) | 125 |
|  | elevated (>1.2) | 22 |
|  |  |  |
| GGT (U/L)^a^ | normal (f: ≤36; m: ≤64) | 85 |
|  | elevated (f: >36; m: >64) | 58 |
|  |  |  |
| CRP (mg/L)^a^ | normal (≤8.2) | 140 |
|  | elevated (>8.2) | 7 |
|  |  |  |
| Diagnosis^a,d^ | primary liver tumors | 67 |
|  | metastases | 82 |
|  |  |  |
| Cholestasis^a,c^ | non-cholestatic | 121 |
|  | cholestatic | 25 |
|  |  |  |

^a^ sample numbers are not summing up to 150 because of missing information

^b^ P450 inducers: atorvastatin, beclometasondipropionate, budenoside, estradiol, metamizole, nifedipine, omeprazole, pantoprazole, prednisolone, simvastatin, tamoxifen, vitamin D

^c^ cholestasis was diagnosed according to Nies et al., 2009

^d^ diagnosis leading to surgery: primary liver tumors included HCC, cholangiocarcinoma, gallbladder tumor; metastases derived from colon carcinomas (65) or from other tumors (17)

Abbreviations: tBili, serum total bilirubin; GGT, serum gamma glutamyl transferse; CRP, C-reactive protein
