# Supplementary figures and images for: A New Panel-Based Next-Generation Sequencing Method for ADME Genes Reveals Novel Associations of Common and Rare Variants With Expression in a Human Liver Cohort
Source: Front Genet. 2019 Jan 31;10:7. doi: 10.3389/fgene.2019.00007 (PMC6365429; doi:10.3389/fgene.2019.00007)

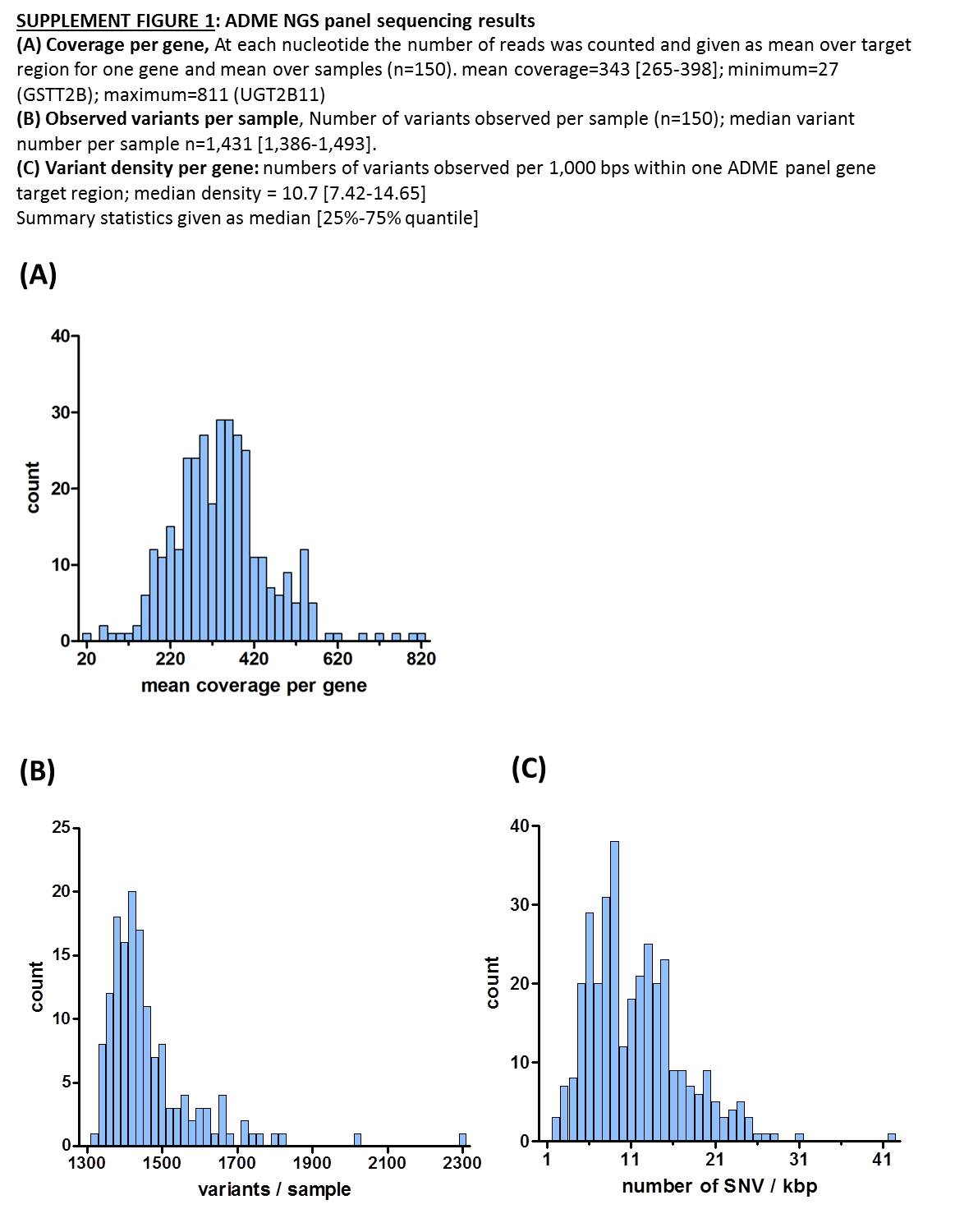

Supplement: Supplementary file 4 [file Image_1.JPEG]

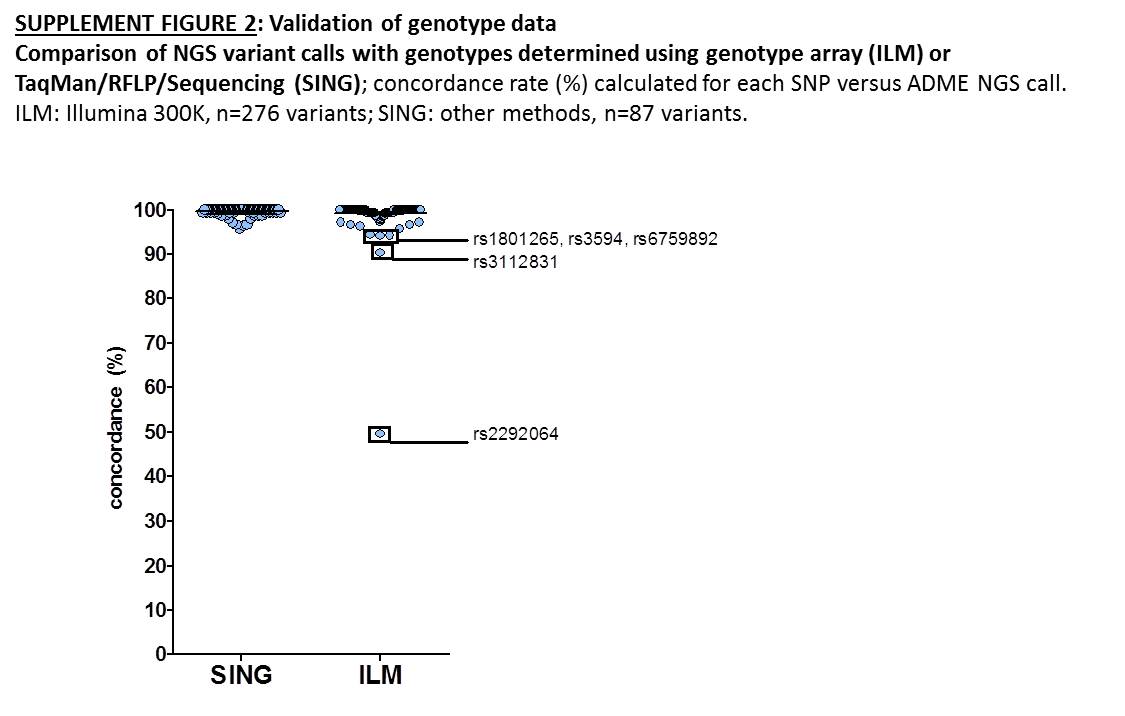

Supplement: Supplementary file 5 [file Image_2.JPEG]

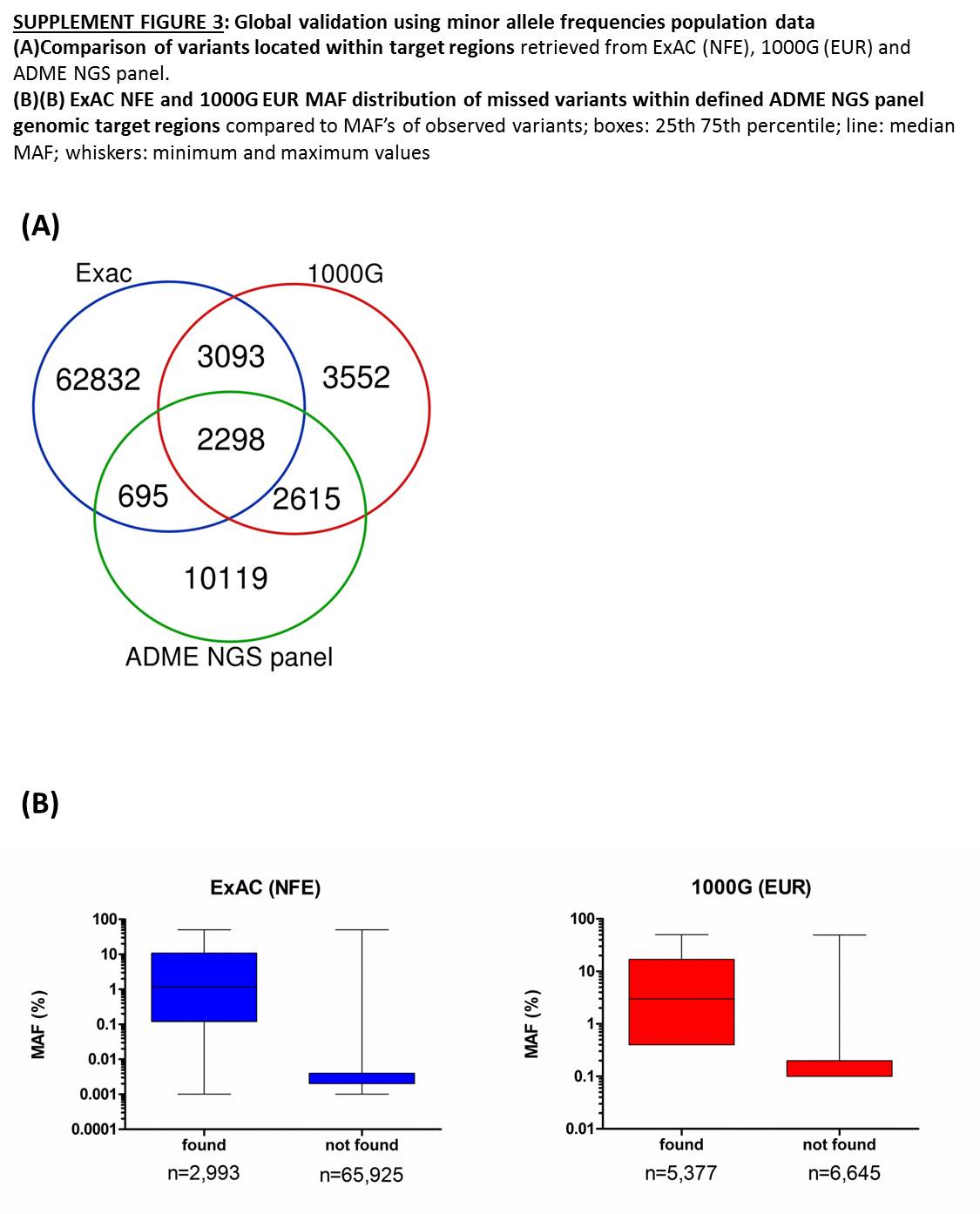

Supplement: Supplementary file 6 [file Image_3.JPEG]
